# Primary care doctors’ views on self-monitoring of blood pressure and self-titration among patients with uncontrolled hypertension in Spain. The ADAMPA trial focus group study.

**DOI:** 10.3399/bjgpopen20X101062

**Published:** 2020-08-19

**Authors:** Irene Marco-Moreno, Patricia Martínez-Ibañez, Eugenia Avelino-Hidalgo, Laura Bellot-Pujalte, Ignacio Barreira-Franch, Lucia Martínez-Ibañez, Marina Escrig-Veses, Margarita Giménez-Loreiro, María Bóveda-García, Mercedes Calleja-del-Ser, Isabel Hurtado, Aníbal García-Sempere, Clara L Rodríguez-Bernal, Salvador Peiró, José Sanfélix-Genovés, Gabriel Sanfelix-Gimeno, the ADAMPA research group

**Affiliations:** 1 Instituto de Investigación Sanitaria INCLIVA, València, Spain; 2 Health Services Research Unit, FISABIO, València, Spain; 3 Red de Investigación en Servicios de Salud en Enfermedades Crónicas (REDISSEC), València, Spain

**Keywords:** hypertension, family medicine, blood pressure self-monitoring, blood pressure self-management, primary health care, qualitative research, focus groups

## Abstract

**Background:**

Despite the increased use of blood pressure (BP) monitoring devices at home, the hypertension of more than 50% of European patients remains uncontrolled. Nevertheless, the self-management of BP, through the combination of home monitoring of BP with self-titration, could be anaccessible and effective tool for improving hypertension control in the primary care setting. The ADAMPA study is a trial with participants randomised to BP self-management (BPSM) with self-titration of antihypertensive medication or to usual care, in a population of patients with poorly controlled hypertension.

**Aim:**

To explore the views and attitudes of primary care doctors participating in the ADAMPA trial regarding BPSM with self-titration.

**Design & setting:**

A focus group study took place with primary care doctors participating in the ADAMPA trial, which was carried out in one health district of the Valencia Health System in Spain.

**Method:**

Nine primary care doctors participating in the ADAMPA trial were included in the focus group. Three researchers (two using manual methods and one using NVivo software) independently conducted a content analysis, reading the transcripts, identifying, classifying, and coding the contents, and developing a conceptual scheme based on these topics.

**Results:**

Participating doctors clearly support home BP monitoring (HBPM), the setting of individual BP targets, and incorporating patient readings into decision-making. They consider it an investment to educate patients for medication self-adjustment and estimate that an important proportion of their patients are potential candidates for hypertension self-management with medication self-titration. However, they show important divergences regarding the role of nursing in BP control.

**Conclusion:**

Primary care doctors participating in the ADAMPA trial feel comfortable with BPSM with self-titration, and would consider extending its use (or the use of some components, such as BP target setting) to other patients with hypertension outside the trial.

## How this fits in

BPSM has been shown to lead to a clinically significant reduction in BP. BPSM interventions whereby patients titrate their own medication according to a pre-arranged plan offers promising evidence of effectiveness, although this is only supported by a few clinical trials, all of which were carried out in the specific environment of the UK NHS. The implementation of BPSM with self-titration depends on acceptance by health professionals in each specific context. This qualitative study is the first to explore primary care doctors' views and attitudes regarding HBPM with self-titration outside the UK, which not only highlights the Spanish doctors' support for this BP management strategy, but also shows important divergences regarding the role of nursing in BP control. Because hypertension is one of the largest contributors to burden of disease and primary care doctors' workload, these views and attitudes are extremely relevant for the design of organisational innovations addressing hypertension management.

## Introduction

While safe and effective antihypertensive pharmacological treatments have been available for decades, hypertension continues to be one of the largest contributors to the global burden of disease.^1^ The degree of hypertension control in Europe has increased over recent years, but more than 50% of the patients treated continue to have BP figures above those recommended in clinical practice guidelines****.^2^ In Spain, a meta-analysis of studies published between 2000 and 2012 shows an overall pooled prevalence of uncontrolled hypertension (>140/90 mmHg) of 67.0%,^3^ ranging from 43.8% to 53.7% in the most recent studies.^4^

The digitalisation of BP monitoring devices, with easier handling and lower prices, has allowed the widespread use of HBPM. Whereas HBPM has some advantages over its measurement in consultation rooms (for example, differentiating between sustained hypertension and white-coat hypertension), systematic reviews do not show a clear advantage for HBPM alone in reducing BP.^5,6^ BPSM, combining HBPM with other co-interventions (such as education, lifestyle counselling, telemonitoring, support from doctors or nurses, systematic titration from doctors or pharmacists, and others), has been shown to lead to a moderate but clinically significant reduction in BP.^6–8^

BPSM interventions, whereby patients titrate their own medication according to a pre-arranged plan, offer limited but promising evidence of effectiveness^9^. For example, two published randomised clinical trials (TASMINH2 ^10^ and TASMINH-SR,^11^ both in the UK) showed systolic BP reductions of 5–9 mmHg versus usual care (a decrease that would translate into a 14% and 9% reduction in stroke and heart disease mortality, respectively^7^) with no increase in side effects,^10,11^ an acceptable cost-effectiveness ratio,^12–14^ and satisfactory acceptance by patients^15–17^ and professionals.^16–18^

The ADAMPA study is a pragmatic trial carried out in Valencia (Spain), with participants randomised to BPSM (with self-titration of antihypertensive medication) or to usual care, in a population of patients with poorly controlled hypertension.^19^ The ADAMPA study included some qualitative sub-studies with patients and professionals (doctors and nurses) to provide an in-depth understanding of the BPSM with self-titration intervention thus far developed, in order to identify the most highly and least valued elements of the intervention, potential barriers and limitations, and possible areas for improvement. The current study aims to explore the views and attitudes of those primary care doctors participating in the ADAMPA trial regarding BPSM with HBPM and self-titration.

## Method

### ​Design

The meeting with primary care doctors participating in the ADAMPA trial was structured according to focus group methodology, which is a qualitative research technique that uses interaction between participants to generate information about their opinions, attitudes, cultures, and values.^20–23^ A descriptive generic approach was used, analysing the themes and patterns that emerged in the narrative content of the meeting.^24^ Supplementary materials (Appendix S1 and S2) incorporate the items included in the consolidated criteria for reporting qualitative research (COREQ) checklist^25^ and the standards for reporting qualitative research (SRQR).^26^

### ​Intervention

The ADAMPA trial is a researcher-initiated, pragmatic, controlled, randomised, non-masked clinical trial with two parallel arms, developed in a Valencia health district (Spain), with the collaboration of 36 family doctors and 13 primary care nurses from 15 primary healthcare centres. The main objective was to evaluate the effectiveness of an intervention based on BPSM (including HBPM, education on arterial hypertension, written BP targets, and self-titration of antihypertensive drugs according to an individualised pre-arranged plan) versus usual care (including education on hypertension but not HBPM training) in patients with poorly controlled hypertension. Patients had to attend consultation within 3 weeks of making any (self)medication changes. Pre-arranged plans did not include dose (self)reductions or treatment dropouts; however, the physician could change the medication in anyway they considered appropriate. The study protocol was registered (https://clinicaltrials.gov/ct2/show/NCT03242785) and has been published elsewhere.^19^ At the time of the focus group meeting, almost all the patients had completed the 12-month predefined period for the main endpoint follow-up, and preliminary results (with 6 months of follow-up) had been presented in one scientific meeting.^27^ The main results of the study had not yet been published, but doctors were aware of improvements of BP control in their own patients included in the trial, and of the 6-months results, which showed a significant improvement over baseline in both intervention and control groups.

### ​Setting

The ADAMPA study was conducted in the Valencia Health System, which is an extensive network of public hospitals and primary healthcare centres. It is part of the Spanish NHS, which is funded and mostly provided by the Valencia regional government, free at the point of care (except for some co-payments for out-of-hospital medication), and virtually universal.^28^ Doctors, nurses, and other healthcare staff enjoy a civil servant-like status, are basically paid by salary, and health care is geographically structured into 'health districts' (the geographical catchment area of one hospital) and 'primary care areas' (the catchment area of one primary healthcare centre).

### ​Recruitment of participants

By consensus within the study coordinators, 12 primary care doctors contributing as collaborating researchers to the ADAMPA trial (one-third of the collaborating doctors) were invited to participate in the focus group. In accordance with the generic descriptive theoretical approach, criterion sampling^24^ was primarily used (the essential selection criterion was participation in the ADAMPA trial), followed by purposive sampling,^24^ which was based on the researchers’ judgement about which potential participants would be most informative, in order to obtain a wide range of variation in backgrounds, views, and attitudes in an attempt to constitute a plural group with different perspectives and from different healthcare centres. The final sample fit the profile of the doctors participating in the trial well (80% women, 90% specialists in family medicine) even if explicit objective selection criteria was not employed, such as sex, age, position, curriculum, or institutional representation. All candidates agreed to participate, but three of them could not attend the focus group meeting: two owing to an unexpected overload of work in their respective centres; and one being out of town. Finally, nine panellists and two coordinators from the research team (JSG, SP) participated in the focus group. Both coordinators were medical doctors with PhD degrees and experience in qualitative research and leadership of focus groups. The participants’ characteristics at the time of the meeting are described in Table 1: most were women (89%) and family medicine specialists (89%), with an average of 23.7 years of work since obtaining their degrees (range 6–36), for the most part in primary care, and from nine different healthcare centres in the city of Valencia (67%) and nearby villages.

**Table 1.** Characteristics of the participants in the focus group

**Sex**(**% women**)	**Family medicine specialist**	**Family medicine residency training**	**Years since medical degree**	**Years in primary care**	**Years in current post**	**Primary care centre**(**% Valencia City**)
***Participants (% or mean)***						
Woman	Yes	Yes	6	3	1	Valencia City
Woman	Yes	Yes	7	3	2	Nearby Villages
Woman	Yes	Yes	20	20	3	Valencia City
Man	Yes	Yes	23	20	3	Valencia City
Woman	Yes	Yes	24	17	3	Nearby Villages
Woman	Yes	Yes	30	24	13	Valencia City
Woman	Yes	Yes	33	25	19	Nearby Villages
Woman	No	No	34	30	13	Valencia City
Woman	Yes	No	36	35	6	Valencia City
**88.9%**	**88.9%**	**77.8%**	**23.7** **years**	**19.7** **years**	**7.0** **years**	**66.7%**
***Coordinators***						
Man	Yes	No	43	38	25	---
Man	No	No	40	0	11	---
***Non-attendants (% or mean)***						
Woman	Yes	Yes	26	20	5	Valencia City
Man	Yes	Yes	33	30	25	Nearby Villages
Woman	Yes	No	34	28	26	Valencia City
**66.7%**	**100%**	**66.7%**	**31.0** **years**	**27.0** **years**	**18.7** **years**	**66.7%**

### ​Focus group dynamics

The meeting, approximately 1 hour long, was held in May 2019 at the headquarters of the Foundation for the Promotion of Health and Biomedical Research of the Valencia Community (FISABIO), a research body under the Valencia government. The research team had previously prepared a list of issues, not previously piloted, to ensure that the most relevant topics were addressed and that discourse saturation was reached (Table 2). Only coordinators and participants were present during the meeting. After the (free) disposition of the participants around the meeting table, the welcome, the participants’ introduction, and the signing of the informed consent forms, the meeting began with a brief explanation of its purpose and the general rules for its development. It highlighted the confidentiality of opinions and the importance of interaction between participants and divergent opinions, expressly pointing out there were no right or wrong answers, and that no attempt would be made to reach a consensus, but rather to explore all opinions and points of view. During the meeting, both moderators adopted an attitude of active and non-judgemental listening, while also leading the discussion towards the topics of interest.^29–31^ The meeting was ended when both moderators considered that all the topics in Table 2 had been explored, the discussion was not providing new themes and the information provided by the group had been exhausted, and participants did not wish to add any new comment. The interview was recorded and transcribed verbatim without identifying individual participants’ opinions.

**Table 2.** Focus group pre-planned questions

Attitudes towards self-monitoring
What proportion of your hypertensive patients do you think have or use BP monitors at home?
Do patients ask you what BP monitor they should buy? Do you recommend the purchase of BP monitors for home use? Do you advise patients on what kind of BP monitor to buy?
Do you offer patients (in general, not in the trial) information on how to take BP at home? When to take it? What to do according to the BP results?
Do you think that HBPM (in real life, outside the trial) contributes to better control of hypertensive patients? Does it help to reduce the 'white-coat syndrome'?
Do you think HBPM reduces follow-up consultations or do you think that patients take BP excessively and that HBPM generates unnecessary consultations?
Do you ask patients to bring their BP monitors to the healthcare centre to compare their readings with those taken in the consultation and check the calibration?
Do you lend BP monitors to patients? (For example, at the time of diagnosis or to have more information before changing a treatment.)
Do you ask patients to bring their readings to the consultation? How do you use those readings? Do you incorporate them into decision making or do you only trust the readings taken during the consultation?
**Trial experience**
How comfortable do you feel about patients having pre-specified medication change plans in advance? Does it produce a higher workload? Do patients also ask before making the pre-specified changes?
Do you consider that many patients would not be eligible for medication self-titration? What proportion of your patients would not be? Which patients would not be suitable candidates?
Do you feel comfortable with this method of pre-specified objectives for changing medication by the patient or would you always prefer to do it in a consultation?
In general terms, do you think your patients have followed the pre-specified medication change instructions?
Have you encountered conflicting situations? For example, has the patient asked why in situations with similar BP readings the doctor has not made treatment changes and now he has to make them? Do patients believe that it is only a way to reduce consultations?
Do patients forget initial training and repeatedly request consultations to ask about self-management?
**Role of self-monitoring and self-adjustment of medication in health care**
Can HBPM with self-titration be a method to reduce the workload of monitoring hypertensive patients? Is the workload for initial training excessive? (Excluding trial activities)
Does HBPM with self-titration offer greater opportunities for patient education and participation in the management of their disease (empowerment)? Is this useful?
In the ADAMPA trial, telemonitoring has not been used. Would you like to implement this type of self-control in an App? Should the App be connected to the electronic medical record or would this be a huge burden of unnecessary readings to evaluate?
What percentage of your patients do you think would adapt to using an App to improve their BP control? Do you think that an App that monitored patients’ BP (for example, a smart watch) would improve their control or generate unnecessary appointments and consultations?
Would you be in favour of introducing HBPM with self-titration as a routine element of hypertension management in primary care?

BP = blood pressure; HBPM = home blood pressure monitoring

### ​

### ​Analysis

Three researchers (IMM, JSG, and SP, two using manual methods and one using NVivo [version 12] software) independently conducted an inductive content analysis, reading the transcripts; identifying, classifying, and coding themes and patterns between and within themes; and developing a conceptual scheme based on these topics as a summary of categories.^24,32,33^ Next, codes were regrouped to resolve the discrepancies by consensus, and finally the contents were classified into four topics: (1) primary care doctors' general views on BPSM; (2) primary care doctors' views on explicit personalised blood pressure targets and self-titration; (3) interprofessional collaboration with nurses; and (4) views on incorporation of new information technologies into the HBPM with or without self-titration. The Results section summarises the information provided by the participants, which is illustrated when appropriate with a selection of literal quotes.

## Results

### ​Primary care doctors’ general views (outside the trial) on self-monitoring at home

Doctors participating in the focus group estimated that half of their patients with hypertension have a BP monitoring device at home, although, this figure may vary according to the socioeconomic level of their primary care area. They do not actively ask patients about BP devices, but on request they recommend the purchase of BP monitors and advise patients on quality brands and devices. On request and in an unstructured way, doctors (or sometimes the nursing staff) offer information on how to take BP and how to interpret the readings:

*'In my primary care area about 40%–50% of hypertensive patients have a BP monitor* […].'

*'In my primary care area, perhaps because it is a district with a high socioeconomic level, more than 50% have one, despite not having hypertension*.'

*'We recommend arm (not wrist) cuffs because they are more reliable* […].'

*'* […] *while we give them the information on how to take blood pressure, we must also inform them how to interpret it* […].'

*'In my case it is the nurse who does it*.'

Doctors ask patients to write readings down and to bring them to appointments. They claim to incorporate this information into treatment decisions, although they worry when there is a significant difference between the BP readings at home and the consultation room. Occasionally, especially if there is a large discrepancy between the readings at home and during the consultation, doctors ask patients to bring the BP monitor to the surgery to verify their calibration.

*'I absolutely insist that they write the readings down* […] *It is not enough to say “my blood pressure is OK”* […].'

*'I provide* [patients] *with a form to write the readings down and with the rules of how BP should be taken* [...] *it requires investing some time but* [...]*'*

*'*[Patients] *are not clear about the concept of “normal”. They do not have high readings but neither are they “normal”, and patients do not have an adequate concept of “proper control”, older people especially. This is the reason why it is important that they bring the readings with them to the surgery*.'

*'What sometimes worries me is the huge difference between the readings they bring and the ones you take at that time* […] *we know there is a white-coat hypertension effect but sometimes there is so much difference* [...] *That’s when we check the device, because of that difference*.'

Primary care doctors think HBPM is very useful for controlling hypertension and reducing the 'white-coat' effect, but also consider that in some patients, especially older people, it occasionally generates doubts and unnecessary consultations and appointments. For a successful HBPM experience, doctors consider it critical to invest time in patient information and training:

*'There are people with a good educational level. They understand everything when you explain things to them. But also, many older people make bad use of home BP monitors and this has caused us many problems. Not one on-call duty shift goes by in which someone does not call us because he/she has woken up at dawn, and he/she has taken their BP* [with abnormal readings].'

*'Very old people who get scared right away. But in general younger people, I think much better*.'

*'There is some educational work* […] *that we have to do* […].'

### ​Primary care doctors’ views on explicit personalised blood pressure targets and self-titration

The self-titration of antihypertensive drugs according to a pre-arranged plan is not a usual practice in primary care outside the ADAMPA study, although it is common to instruct the patient to reduce antihypertensive medication as a result of low BP in specific situations such as hot summers. Participating doctors were enthusiastic about setting individual targets and explaining them to patients, and have applied this self-management component to many of their patients regardless of the self-titration component. Likewise, doctors feel very comfortable with patients’ self-titration and do not consider it to be different from self-titration in diabetes, although they sometimes worry that the patient may misunderstand the instructions and abandon or reduce treatment:

*'Only some of them intuitively and others because you have warned them: if blood pressure drops due to heat or because they are dieting or losing weight* ...'

*'It gives them security. Nervousness and anxiety decrease. They are no longer so obsessed, look more confident and can participate: “I no longer have to go to the doctor so many times.* *“* *'*

*'It gives us security. You can set a goal for each patient and that also gives them security. Knowing what to do. It’s the same as blood glucose*.'

Regarding self-titration, doctors believe there is a form of therapeutic inertia in patients, which leads them to not increase medication even if they are above their BP targets. Nevertheless, they consider this behaviour varies from one patient to another, and one of their tasks is to select the monitoring method that best suits each patient:

*'There are many patients who do not adjust their medication when they are well above their BP objective until they consult you*.'

*'If it is a small increase, for example, an increase of half a tablet, they do it. But not if they have to introduce a new medication. They are afraid of being aggressive with the treatment*.'

*'There are many types of patients and generalising is very complex.* […] *There are patients for whom these techniques are great and other patients for whom these techniques may be counterproductive. As we know our patients, we know which patient will do very well and who will not*.'

Doctors have differing views over the percentage of potential patients who would be candidates for self-titration. For some they would be useful only for some patients, while for others self-titration would be possible in almost all patients:

*'Self-adjustment I think would depend on the patient* [several doctors showing agreement with this answer].'

*'Not everyone is a candidate* […]*'*

*'I think everyone is a potential candidate. Older people made a mess at the beginning, but in the end they have greatly improved their self-control*.'

*'Everybody. Even if they don't handle it well all the time*.'

### ​Interprofessional collaboration

During the meeting, differences regarding the current role of primary care nurses in the control of BP emerged (from being of no value or limited value to helping reduce medical appointments, to being essential for the management of hypertension). There seems to be a great heterogeneity between centres (even between individual professionals) in the role played by nurses in the management of patients with hypertension, and also in the quality of the interprofessional collaboration between doctors and nurses:

*'As more and more people have BP monitors at home, they have stopped going to nursing consultations.* [Patients] *save themselves the nursing control consultations that, otherwise, didn’t serve any purpose*.'

*'*[Nurses] *take BP but do not do any educational activity, so I prefer patients to do it at home*.'

*'What I try with my nurse is to optimise appointments: instead of coming every month, patients come every 6 months*.'

*'Self-adjustment and HBPM must always be reinforced in each patient with the support of nursing. I think it is much more productive to do a good part of hypertension management in nursing consultations. Having good nursing support is essential*.'

*'Nursing support for hypertension control has been lost. This support would be fantastic, but with my hypertensive patients I don’t count on nursing staff at all*.'

*'There is also nursing inertia. In patients, but in nurses as well. They’ve seen several high readings but have not recommended them to see their doctor. Consequently, we keep patients in the range of 140–150 mmHg who shouldn't be in that range*.'

*'Having good nursing support is essential* [several participants agreed with this answer].'

### ​Primary care doctors’ views on the incorporation of new information technologies into the HBPM with or without self-titration

Doctors point out that many patients, especially the youngest ones, already go to the appointment with their tablet and their BP readings (or weight, physical exercise, blood glucose readings, and so on) annotated, but electronic systems would not be suitable for all patients, especially the oldest:

*'A lot of people demand it from you: "Could I send them to you by e-mail?" …'*

*'I have a couple of patients in the ADAMPA study who, in addition to the study’s notebook, bring me their own notebook with everything pointed in it: “Look my own notebook, doctor, I don’t get used to your booklet.”*'

*'There are people who come with apps or with the tablet. And people that graph their BP in the tablet*.'

Doctors were very favourable towards patient readings at home being automatically incorporated into the electronic medical record. In fact, there are patients who ask them if they can send readings by email in order to avoid follow-up appointments, but doctors are concerned about the possible increase in workload with a massive sending of data (which is often of no clinical value) without an automated analysis strategy for filtering relevant or urgent information. On the other hand, they distrust the information provided by internet or self-care apps, and they worry about the potential for medical over-control with new technologies:

*'Internet and applications often generate more medical consultations but worse information. They are worse quality consultations because you also have to refute what the internet or application has said*.'

*'Professionals should be trained in the first place. Apps have been launched into the general population but professionals have not been informed, and now patients start asking questions. And you also wonder: what is its reliability? How does it work? How do I handle it? What strategies should I follow?'*

*'With all that, will we not be promoting excessive BP control?'*

## Discussion

### ​Summary

One of the most remarkable results of the focus group is the ease with which primary care doctors participating in the ADAMPA trial accepted a hypertension BPSM intervention that included self-titration. It is an unusual component in clinical practice (except in the management of insulin-dependent diabetes or oral anticoagulation), which has only been supported by a few clinical trials carried out in the specific environment of the UK NHS.^10,11^ Briefly, participating doctors in the ADAMPA trial clearly support HBPM, the setting of individual BP targets, and the incorporation of patient readings into decision-making. They consider it an investment to educate patients on medication self-adjustment and, even with divergences, estimate that a significant proportion of their patients are potential candidates for BPSM with antihypertensive drug self-titration. Surprisingly, while participants recognise the importance of information and the training of patients in self-management, they do not seem to have developed this activity in a systematic and structured way which, outside the trial protocol, seems to be carried out sporadically or only on patient request.

Structured nursing programmes or activities dedicated to HBPM or BPSM that are properly coordinated with primary care doctors do not seem to exist in this setting, with some participants minimising the current role of primary care nurses in training and controlling patients with hypertension, while others consider their role to be fundamental. These divergences are probably related to the inadequate organisational design of interprofessional collaboration in primary care in the Spanish NHS. This improper design has been generating a conflict between doctors and nurses, producing a great heterogeneity between primary healthcare centres in the role played by both professions in the care of chronic patients, and the analysis and search for solutions has been postponed.

In relation to the incorporation of new technologies into the management of hypertension in primary care, participants show a certain lack of awareness of integrating HBPM data into the electronic medical record, although some doctors who have used this type of application consider them useful. The acceptance of these technologies probably depends on a user-friendly design that allows the efficient integration of readings and other patient information into the electronic medical record without overloading or interrupting the professional workflow.^34^

### ​Strengths and limitations

The main study limitation, directly related to the extrapolation of its conclusions to all Valencia Health System primary care doctors, is that doctors collaborating in an unpaid independent trial could be different from non-participants. Additionally, ADAMPA trial participants received diverse information about BPSM, BP guidelines, the ADAMPA protocol, and intermediate results at 6 months, and were also aware of the improvement of BP control in their own patients. Study participation, information, and self-perceived better results with their own patients may have influenced their opinions and attitudes, resulting in the modelling of different perspectives than those of the wider population of primary care doctors. Second, the study reports here the views of the primary care doctors who participated in the ADAMPA trial, but not all relevant perspectives on BPSM with self-titration in primary care. The authors are currently developing qualitative studies with nurses and patients participating in the trial in order to provide a more comprehensive portrait. Other potential limitations, common in qualitative studies, include that coordinators of the meeting were researchers on the ADAMPA trial, an aspect that could incorporate a 'social desirability' bias, and the selection (and self-selection, owing to non-attendance of three candidates) of participants which, although there was an attempt to make them as varied as possible, could have reduced the presence of participants more critical of the self-titration component.

### ​Comparison with existing literature

The favourable views of participant doctors are consistent with a recent survey of more than 2000 Spanish primary care doctors, 67% of whom recommend HBPM 'usually' or 'always'.^35^ This survey also found 'modest levels of availability and utilisation' of BPSM and HBPM. This is also consistent with the paradoxical absence of systematic and structured BPSM activities, despite the favourable opinions and attitudes shown in the focus group. A qualitative study using semi-structured interviews with healthcare professionals participating in the TASMINH2 trial shows similar favourable opinions about BPSM with self-titration, but UK professionals, interviewed 5 years before the current study (at a time with lower use of HBPM and BPSM), were more cautious about their implementation. The UK professionals suggested that more information about 'how to train patients to measure blood pressure and how home readings become part of their care' was needed before BPSM with self-titration could be widely implemented.^18^

### Implications for practice

The potential implications of BPSM strategies for hypertension management, for primary care, and for the health system as a whole are considerable. Some studies show control of hypertension consultations accounts for around 10% of all medical appointments, with most consultations occurring in primary care.^36,37^ Therefore, primary care doctors’ views and attitudes are extremely relevant for the design of organisational innovations addressing hypertension management. The focus group in this study demonstrates that primary care doctors participating in the ADAMPA trial feel comfortable with self-management with self-titration, and would consider extending its use (or the use of some components such as BP target setting) to other patients with hypertension outside the trial. They do, however, anticipate more difficulties with older people, who make up a substantial proportion of patients with hypertension.

## Trial registration number

EudraCT 2016-003986-25; https://www.clinicaltrialsregister.eu/ctr-search/search?query=2016-003986-25) and clinicaltrials.gov (NCT03242785; https://clinicaltrials.gov/ct2/show/NCT03242785
